# Reproducibility and diurnal variation in middle cerebral artery blood velocity in healthy humans

**DOI:** 10.1113/EP090873

**Published:** 2023-03-23

**Authors:** Brian Shariffi, Iman N. Lloyd, Mikala E. Cessac, Jennifer L. Harper, Jacqueline K. Limberg

**Affiliations:** ^1^ Department of Nutrition and Exercise Physiology University of Missouri Columbia Missouri USA

**Keywords:** blood flow, brain, chronotype

## Abstract

Transcranial Doppler (TCD) is used to assess cerebral blood velocity (CBV) and cerebrovascular reactivity (CVR). Assessments of TCD reproducibility are limited, and few include multiple within‐day measurements. We sought to establish reproducibility of CBV and CVR in healthy adults during three time periods (morning, afternoon and evening). We hypothesized that CBV and CVR measured at the same time of day are reproducible between days. We also hypothesized that CBV and CVR exhibit diurnal variation, with measurements being higher in the evening compared with morning/afternoon hours. Twelve adults [six male and six female, 27 years (95% CI, 22–31 years)] completed three measurements (morning, afternoon and evening) on two separate days in controlled conditions (e.g., meals, activity and sleep). Middle cerebral artery blood velocity (MCAv, TCD) was measured continuously at rest and during two CVR tests (end‐expiratory apnoea and carbogen inhalation). Intraclass correlation coefficients for resting MCAv showed moderate to good reproducibility, which did not differ between morning, afternoon and evening (0.87, 0.56 and 0.67, respectively; *P* > 0.05). Intraclass correlation coefficients for peak MCAv during apnoea (0.80, 0.46 and 0.65, respectively; *P* > 0.05) and minute 2 of carbogen inhalation (0.81, 0.74 and 0.73, respectively; *P* > 0.05) were also not different from morning compared with afternoon/evening. Time of day had no effect on resting MCAv (*F* = 0.69, *P* = 0.51, ƞ_p_^2^ = 0.06) or the peak response to apnoea (*F* = 1.00, *P* = 0.39, ƞ_p_^2^ = 0.08); however, peak MCAv during carbogen breathing exhibited diurnal variation, with highest values in the evening (*F* = 3.41, *P* = 0.05, ƞ_p_^2^ = 0.24). Measures of CBV and CVR assessed via TCD during morning, afternoon and evening hours are reproducible between days. There is diurnal variation in the MCAv response to carbogen exposure, with CVR being highest during evening compared with morning hours.

## Abstract (highlights)

- **What is the central question of this study?**
  We sought to establish between‐day reproducibility in estimates of middle cerebral artery blood velocity (MCAv) and cerebrovascular reactivity (CVR) in young, healthy male and female adults in tightly controlled experimental conditions.
- **What is the main finding and its importance?**
  Measures of MCAv assessed during morning, afternoon and evening hours are reproducible between days. There is diurnal variation in CVR, with values being highest during the evening compared with the morning. Greater diurnal variation in CVR is associated with more efficient sleep and greater nocturnal blood pressure dipping. These data enhance our understanding of modulators of MCAv and CVR.

## 1 INTRODUCTION

Transcranial Doppler (TCD) is a non‐invasive tool used for identification and monitoring of underlying cerebrovascular conditions and diseases, such as stroke and traumatic brain injury (Fatima et al., 2019; Sarkar et al., 2007). TCD can be used to assess cerebral blood velocity, cerebral autoregulation, cerebrovascular reactivity (CVR), intracranial pressure, cerebral perfusion pressure and other indicators of cerebral haemodynamics (Cardim et al., 2016; Naqvi et al., 2013; Pan et al., 2022; Purkayastha & Sorond, 2012). Although TCD has been widely used in research, clinical and outpatient settings, controversy remains regarding its application, because studies specifically designed for unbiased reproducibility of TCD assessments are limited (Zhao et al., 2022). In studies that have assessed reproducibility of TCD measurements, few have conducted measurements at different time points throughout the day (Strohm et al., 2014), and they are primarily restricted to morning assessments only (Demolis et al., 1993; Mayberg et al., 1996). Furthermore, many have failed to account for the potential impact of sleep, physical activity, diet and/or menstrual cycle phase on measurement reproducibility. Prior work has also focused predominantly on resting measurements (Demolis et al., 1993; Kaczynski et al., 2018), rather than indices of CVR, which are more indicative of overall cerebrovascular health (Catchlove et al., 2018).

Measures of cerebral haemodynamics as assessed by TCD have been shown by several groups to vary throughout the day. For example, CVR to carbon dioxide (CO_2_) or breath holding (apnoea) was found to be at its peak in the evening hours (18.00–21.00 h) and lower in the early morning (06.00–08.00 h) (Ainslie et al., 2007; Ameriso et al., 1994; Conroy et al., 2005; Cummings et al., 2007; Qureshi et al., 1999), although controversy exists (Strohm et al., 2014). Others have shown circadian oscillations in resting middle cerebral artery blood velocity (MCAv) to exhibit a nadir mid‐day (11.00–12.00 h) (Conroy et al., 2005; Demolis et al., 1993; Diamant et al., 2002). The diurnality of cerebrovascular haemodynamics has been hypothesized to contribute, at least in part, to cerebral ischaemia and stroke, which occur predominantly in the morning hours (Elliott, 1998; Etsuda et al., 1999; Wroe et al., 1992). However, in prior studies that have assessed reproducibility of TCD measures, few have conducted multiple measurements throughout the same day nor have they measured and/or controlled for important factors known to impact cerebral haemodynamics (i.e., sleep, physical activity, meals and menstrual cycle) (Caldwell et al., 2021; Hajjar et al., 2007; Pereira et al., 2022; Qureshi et al., 1999; Wauschkuhn et al., 2005). Appreciating diurnal variation in MCAv and controlling for known modulators of cerebral blood velocity is essential to ensure reliability and reproducibility of the measurements, ultimately improving interpretation and translation to real‐life application.

The present investigation sought to establish the between‐day reproducibility in estimates of MCAv and CVR in young, healthy male and female adults. We conducted measurements of MCAv and CVR at three individual time points (morning, afternoon and evening) on two separate days in tightly controlled experimental conditions, including controlled meals, physical activity, menstrual cycle phase and measurement of sleep quality and 24 h blood pressure. We hypothesized that MCAv and CVR measured at the same time of day would be reproducible between days. We also hypothesized that MCAv and CVR would exhibit diurnal variation, with higher values observed in the evening and lower values in the morning and afternoon hours.

## 2 METHODS

### 2.1 Participants

All experiments and procedures were approved by the Institutional Review Board at the University of Missouri (#2016225), were in accordance with institutional guidelines and conformed to the *Declaration of Helsinki* except registration in a database. All participants (*n* = 12, six males and six females) were young (<45 years of age), non‐obese (body mass index < 30 kg/m^2^) and non‐smokers. Participants did not carry a chronic disease diagnosis and were taking no medications. Female participants were premenopausal, had natural menstrual cycles and were studied in the self‐reported early follicular phase of the menstrual cycle {day of menses, visit 1, 6 [95% confidence interval (CI), 4–8]; visit 2, 8 (95% CI, 5–10)}. Females were required to have a negative urine pregnancy test to proceed with the study protocol. All participants were instructed to abstain from alcohol, caffeine, non‐steroidal anti‐inflammatory drugs and strenuous physical activity for 24 h before study visits, according to recently published guidelines (Limberg et al., 2020).

Informed consent was obtained from all participants on a screening visit, followed by medical history, baseline anthropometrics, resting blood pressure, completion of the Berlin (Chiu et al., 2017) and Morningness‐Eveningness (Horne & Ostberg, 1976) questionnaires, and familiarization with TCD. Participants were given a 24 h ambulatory blood pressure monitor (Mobil‐O‐Graph; IEM) and Actiwatch (Phillips Respironics) for continuous measurements of physical activity, in addition to sleep duration and efficiency, using proprietary software (Actiware). During the testing period, participants were asked to limit physical activity/avoid strenuous exercise and aim for 7–9 h of sleep. An overnight pulse oximeter was worn during sleep, with oxygen saturation and heart rate monitored continuously for calculation of the oxygen desaturation index (Wrist Ox_2_; Nonnin).

All participants completed three visits per day (morning 06.00–08.00 h, afternoon 12.00–14.00 h and evening 18.00–20.00 h) on two separate days (visits 1 and 2) within 1 week. All visits were completed in the fasted state. A standardized meal was provided on the night before the first study visit and after every subsequent visit. Total calories were calculated using the Harris–Benedict equation and distributed into a macronutrient breakdown of 45% carbohydrate, 35% fat and 20% protein (Manore, 2005).

### 2.2 Study visit

Participants arrived at the laboratory fasted, and they rested supine in a dimly lit room for equipment instrumentation. Individuals were instrumented with a three‐electrode ECG to measure heart rate (lead II; Bio Amp FE132; ADInstruments) and continuous blood pressure measurement by finger photoplethysmography (Human NIBP Controller ML282; ADInstruments) calibrated to upper arm blood pressure. Oxygen saturation was monitored by finger pulse oximetry (Oximeter Pod, Fingerclip ML320/F; ADInstruments) and respiration by use of a piezo respiratory belt transducer (MLT1132; ADInstruments). Participants wore a face mask covering the nose and mouth and connected to a non‐rebreathing valve for the duration of the testing period. Because wearing a face mask might affect normal breathing and introduce the potential for increased dead space breathing compared with a standard mouthpiece, mask size was documented, and the same mask was used for each study visit. Breath‐by‐breath tidal volume and respiratory rate were measured with a pneumotachograph (Hans Rudolph, Shawnee, KS, USA) and a differential pressure amplifier (PA‐1, Series 1110; Hans Rudolph). Inspired and expired gases (Gemini 14–10000 Respiratory Monitor; CWE) were monitored continuously.

MCAv was measured continuously using TCD ultrasonography. The left middle cerebral artery was insonated using a 2 MHz Doppler probe (Multigon TOC Neurovision Transcranial Doppler, Elmsford, NY, USA) following published guidelines (Willie et al., 2011). The same sonographer (B.S.) adjusted the Doppler probe over the transtemporal window located superior to the zygomatic arch. After the signal was identified at the appropriate depth (range 40–55 mm), the probe was fixed securely using a headpiece to prevent movement and maintain signal quality. The middle cerebral artery was located and verified using the parameters of depth, directionality of the signal in relationship to the probe, and signal response. Signal depth, gain and amplitude were noted in a log for each study participant. A small mark was also made on the skin at the location of the probe using a permanent marker, and this location was used for repeat visits. Mean MCAv was normalized for mean arterial blood pressure (MAP) [MCAv ÷ MAP × 100] for measures of cerebrovascular conductance index (CvCi).

### 2.3 Protocol

After instrumentation, participants were asked to keep their eyes open during a 10 min quiet resting period. After quiet rest, CVR was assessed during two 20 s end‐expiratory apnoeas, each of which was followed by a 2 min washout to ensure haemodynamic variables returned to baseline (Przybyłowski et al., 2003; Tancredi & Hoge, 2013). Participants then completed 2 min of quiet rest while breathing room air, followed by inhalation of carbogen (5% carbon dioxide and 95% oxygen) for 2 min (Burley et al., 2020; McDonnell et al., 2013; Totaro et al., 1999), with a 50 L meteorological balloon serving as a volume reservoir.

### 2.4 Data analysis

All data were recorded at 1000 Hz using a computer data acquisition system (PowerLab; ADInstruments) and stored for offline analysis. Baseline data are reported as an average of the last 90 s of the 10 min quiet rest. Results from the apnoea trials were analysed second by second during apnoea and throughout a 10 s recovery (Cummings et al., 2007; Przybyłowski et al., 2003). Data were assessed as a 3 s rolling average, results from the two apnoea trials were averaged, and the peak response was determined. Results from the carbogen protocol were analysed second by second, and data are reported as a 60 s average from the second minute and as a 1 s peak (Burley et al., 2020; Carr et al., 2021). Absolute changes in MCAv from baseline during apnoea and carbogen trials were calculated (Δ = intervention minus baseline). Measures of CVR were calculated as the ratio of a change in MCAv (Δ, in centimetres per second) with a change in carbon dioxide (Δ, in millimetres of mercury) during carbogen trials.

### 2.5 Statistical analysis

Statistical analysis was completed using SigmaPlot v.14.0; (Systat Software). Between‐day variability was determined in three ways. First, the coefficient of variation (SD/mean × 100) was calculated. Second, the response relationship over the two separate days was compared using linear regression analysis and Pearson product–moment correlation. Third, differences observed between the two test sessions were plotted against the mean value of the two tests (Bland–Altman method of differences). The 95% limits of agreement were calculated. Linear regression analysis and Pearson product–moment correlation were then applied to the Bland–Altman plot to assess proportional bias. To characterize reproducibility further, intraclass correlation coefficients (ICCs) were calculated using a mixed effects model (Blanca et al., 2017; Koo & Li, 2016). Differences in ICC by time of day were assessed using a single‐sided test (Eid et al., 2010).

To determine the effect of time of day (i.e., diurnal variation) and/or trial time (e.g., percentage apnoea time) on main outcome variables, a one‐ or two‐way repeated‐measures ANOVA was applied, respectively. Normality was assessed using the Shapiro–Wilk test, and multiple comparisons were made with the Holm–Sidak method. Non‐parametric tests (Kruskal–Wallis one‐way ANOVA on ranks) were used when necessary, and multiple comparisons were made with the Bonferroni *t*‐test. Effect size is reported as ƞ_p_^2^ [(SS_treatment_/(SS_treatment_ + SS_error_)], where SS stands for sum of squares. In a post hoc analysis, the response relationship between diurnal variation of CVR during carbogen breathing (evening minus morning) and participant characteristics (e.g., age, body mass index, chronotype and sleep duration) were explored using linear regression analysis and Pearson product–moment correlation. Data are reported as the mean (95% CI), and significance was determined as *P* ≤ 0.05.

We calculated the required sample size for CVR a priori based on the ability to detect a 0.60 ± 0.60%/mmHg difference in CVR, with the standard deviation based on data from Ainslie et al. (2007). Using α = 0.05 and two‐sided testing, this resulted in a total sample size of 10 participants to achieve power of 0.80. Likewise, data from Ameriso et al. (1994) supported the need for nine participants to detect a 0.62 ± 0.56%/mmHg difference in CVR with power of 0.80 and α = 0.05. Using this information as a guide, we recruited 12 healthy young adults, with an equal number of male and female participants.

## 3 RESULTS

Twelve healthy adults [six male and six female, 27 years of age (95% CI, 22–31) and with body mass index of 25 kg/m^2^ (95% CI, 23–27)] participated in the present investigation. All participants were classified as low risk for having obstructive sleep apnoea via both the Berlin questionnaire (low risk, *n* = 12) and oxygen desaturation index [1.1 events/h (95% CI, 0.8–1.5), range 0.4–2.5]. All participants had blood pressures within normal ranges on the screen visit [116 mmHg (95% CI, 112–121)/71 mmHg (95% CI, 67–75)]. Participants were categorized via the Morningness‐Eveningness Questionnaire as moderate morning (*n* = 5, two females and three males), intermediate (*n* = 5, two females and three males) or moderate evening (*n* = 2, two females and no males) chronotype.

### 3.1 Reproducibility

Sleep, diet and physical activity were well controlled between visits (Table 1). Resting MCAv exhibited moderate to good reproducibility (ICC, morning 0.87, afternoon 0.56 and evening 0.67). The ICC for resting MCAv did not differ between morning, afternoon and evening hours (*P* range = 0.07–0.35). Linear regression analysis also supported low between‐day variability in resting MCAv (morning, *r* = 0.93, *P* < 0.01; afternoon, *r* = 0.65, *P* = 0.02; evening, *r* = 0.66, *P* = 0.18). Proportional bias was not observed when linear regression was applied to the Bland–Altman plot (morning, *r* = 0.06, *P* = 0.85; afternoon, *r* = 0.33, *P* = 0.30; evening, *r* = 0.18, *P* = 0.57; Table 2).

**TABLE 1:** Between‐day variability in lifestyle factors.

Factor	Day 1	Day 2
Sleep		
Sleep duration (min)	409 (376, 442)	435 (410, 459)
Sleep efficiency (%)	91 (89, 93)	91 (89, 93)
Diet		
Total kilocalories	1912 (1728, 2097)	1916 (1732, 2099)
Carbohydrates, %	45 (45, 46)	46 (45, 46)
Fats, %	36 (35, 37)	36 (35, 37)
Proteins, %	21 (20, 21)	21 (20, 21)
Physical activity, % daily activity		
Sedentary	55 (48, 61)	54 (48, 60)
Low	25 (21, 29)	28 (22, 33)
Low–moderate	12 (10, 14)	11 (8, 13)
Moderate	6 (6, 7)	6 (5, 7)
Moderate–vigorous	2 (1, 2)	2 (1, 2)
Vigorous	0 (0, 0)	0 (0, 0)
Blood pressure, mmHg		
24 h SBP	117 (114, 120)	117 (113, 121)
24 h DBP	72 (69, 75)	71 (67, 75)
24 h MBP	93 (91, 95)	92 (89, 95)
Daytime SBP	119 (116, 122)	119 (115, 123)
Daytime DBP	74 (71, 78)	73 (69, 77)
Daytime MBP	95 (92, 98)	94 (91, 98)
Nighttime SBP	109 (105, 112)	107 (102, 112)
Nighttime DBP	64 (62, 67)	63 (59, 66)
Nighttime MBP	85 (82, 87)	83 (79, 87)

*Note*: Data are presented as the mean (95% confidence interval) and were analysed using a one‐way repeated‐measures ANOVA unless otherwise noted (repeated‐measures ANOVA on ranks: % carbohydrates, % fats, % proteins, total kilocalories and low–moderate). Data are from *n* = 12 unless otherwise noted (nighttime blood pressure, *n* = 10 or 11).

*P* > 0.05 for all.

Abbreviations: DBP, diastolic blood pressure; MBP, mean blood pressure; SBP, systolic blood pressure.

**TABLE 2:** Between‐day repeatability outcomes by regression analysis and the Bland–Altman method of differences.

			Pearson correlation		Bland–Altman		
Variable	% CV	ICC	*r*	*P*‐value	95% Confidence interval	*r*	*P*‐value
Resting MCAv, cm/s							
Morning	8 (4, 11)	0.869	0.931	<0.01	−8.3, −2.5	0.063	0.85
Afternoon	13 (7, 19)	0.557	0.654	0.02	−0.2, 14.5	0.329	0.30
Evening	11 (7, 16)	0.670	0.664	0.18	−4.1, 7.8	0.183	0.57
Peak apnoea MCAv, cm/s							
Morning	7 (4, 11)	0.801	0.818	<0.01	−8.8, 1.0	0.054	0.87
Afternoon	12 (6, 17)	0.459	0.458	0.13	−4.6, 12.8	0.102	0.75
Evening	11 (6, 16)	0.654	0.642	0.02	−5.7, 9.6	0.110	0.73
∆Peak apnoea MCAv, cm/s							
Morning	25 (18, 33)	0.708	0.720	<0.01	−1.7, 4.0	0.315	0.32
Afternoon	36 (16, 57)	0.275	0.334	0.29	−8.7, 0.0	0.311	0.33
Evening	29 (16, 43)	0.368	0.355	0.26	−4.8, 4.9	0.215	0.50
Minute 2 mixed gas MCAv, cm/s							
Morning	7 (4, 11)	0.813	0.881	<0.01	−9.7, −1.9	0.219	0.49
Afternoon	11 (8, 14)	0.739	0.755	<0.01	−2.9, 10.2	0.275	0.39
Evening	10 (5, 16)	0.728	0.714	<0.01	−7.0, 7.0	0.153	0.63
∆Minute 2 mixed gas MCAv, cm/s							
Morning	33 (12, 54)	0.604	0.608	0.04	−3.3, 1.0	0.072	0.82
Afternoon	54 (23, 86)	0.376	0.449	0.14	−7.2, −0.5	0.171	0.60
Evening	45 (27, 63)	0.122	0.116	0.72	−7.1, 4.0	0.143	0.66
Minute 2 mixed gas reactivity, cm/s/mmHg							
Morning	34 (19, 49)	0.519	0.642	0.02	−0.8, −0.1	0.353	0.26
Afternoon	33 (15, 51)	0.384	0.508	0.09	−0.9, −0.2	0.259	0.42
Evening	40 (20, 60)	0.215	0.232	0.47	−1.1, 0.2	0.247	0.44
Peak mixed gas MCAv, cm/s							
Morning	8 (4, 12)	0.777	0.860	<0.01	−11.6, −2.7	0.087	0.79
Afternoon	10 (7, 13)	0.753	0.777	<0.01	−3.1, 10.6	0.355	0.26
Evening	10 (6, 14)	0.717	0.706	0.01	−8.3, 7.0	0.194	0.55
∆Peak mixed gas MCAv, cm/s							
Morning	18 (8, 28)	0.596	0.657	0.02	−5.2, 0.1	0.278	0.38
Afternoon	28 (13, 44)	0.446	0.537	0.07	−7.5, 0.0	0.418	0.18
Evening	21 (12, 31)	0.148	0.145	0.65	−7.4, 3.0	0.081	0.80
Peak mixed gas MCAv reactivity, cm/s/mmHg							
Morning	24 (15, 34)	0.332	0.401	0.20	−1.2, 0.0	0.260	0.41
Afternoon	20 (10, 30)	0.379	0.410	0.19	−0.8, 0.3	0.423	0.17
Evening	22 (10, 33)	0.421	0.515	0.09	−1.2, 0.1	0.523	0.08

*Note*: Data are presented as the mean (95% confidence interval) from *n* = 12 participants. Linear regression (ordinary least squares regression analysis) was applied to the Bland–Altman plot to assess proportional bias.

Abbreviations: CV, coefficient of variation; ICC, intraclass correlation coefficient; MCAv, middle cerebral artery blood velocity.

The MCAv observed at peak apnoea exhibited moderate to good reproducibility (ICC: morning 0.80, afternoon 0.46 and evening 0.65). The ICC for peak MCAv during apnoea did not differ between morning, afternoon and evening hours (*P* range = 0.10–0.27). Linear regression analysis also supported low between‐day variability in MCAv at peak apnoea (morning, *r* = 0.82, *P* < 0.01; afternoon, *r* = 0.46, *P* = 0.13; evening, *r* = 0.64, *P* = 0.03). Proportional bias was not observed when linear regression was applied to the Bland–Altman plot (morning, *r* = 0.05, *P* = 0.87; afternoon, *r* = 0.10, *P* = 0.75; evening, *r* = 0.11, *P* = 0.73; Table 2).

The MCAv observed during minute 2 of carbogen exposure exhibited moderate to good reproducibility (ICC: morning 0.81, afternoon 0.74 and evening 0.73). The ICCs for MCAv at minute 2 of carbogen breathing were not different from morning compared with afternoon/evening (*P* range = 0.33–0.48). Linear regression analysis further supported low between‐day variability in MCAv at minute 2 of carbogen exposure (morning, *r* = 0.88, *P* < 0.01; afternoon, *r* = 0.75, *P* < 0.01; evening, *r* = 0.71, *P* = 0.01). Proportional bias was not observed when linear regression was applied to the Bland–Altman plot (morning, r = 0.22, *P* = 0.49; afternoon, *r* = 0.28, *P* = 0.39; evening, *r* = 0.15, *P* = 0.63; Table 2).

Measures of CVR calculated as the ratio of a change in MCAv with a change in carbon dioxide during minute 2 of carbogen breathing exhibited relatively poor reproducibility (ICC: morning 0.52, afternoon 0.38 and evening 0.21); similar conclusions were drawn when assessed using the peak change in MCAv (ICC: morning 0.33, afternoon 0.38 and evening 0.42). Linear regression analysis showed low between‐day variability in morning CVR (minute 2, *r* = 0.64, *P* = 0.02; peak, *r* = 0.40, *P* = 0.20); however, when carbogen breathing occurred in the afternoon (minute 2, *r* = 0.51, *P* = 0.09; peak, *r* = 0.41, *P* = 0.19) and evening (minute 2, *r* = 0.23, *P* = 0.47; peak, *r* = 0.51, *P* = 0.09), results exhibited variability between days. Notably, proportional bias was not observed when linear regression was applied to the Bland–Altman plot (minute 2: morning, *r* = 0.35, *P* = 0.26; afternoon, *r* = 0.26, *P* = 0.42; evening, *r* = 0.25, *P* = 0.44; peak: morning, *r* = 0.26, *P* = 0.41; afternoon, *r* = 0.42, *P* = 0.17; evening, *r* = 0.52, *P* = 0.08; Table 2).

### 3.2 Diurnal variation

**FIGURE 1:**
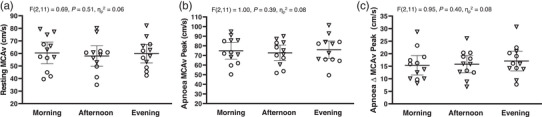
Diurnal variation in resting middle cerebral artery blood velocity (MCAv) and the peak response to apnoea. Data are presented as the mean and 95% confidence interval and as individual data points from *n* = 12 participants [six males (open circles) and six females (open inverted triangles)]. Statistical analysis was conducted using a one‐way repeated‐measures ANOVA. There was no effect of time of day on resting MCAv (a) or on the peak MCAv response to apnoea (b,c).

Meal composition varied within the day, with more carbohydrates and less fats/proteins consumed after the morning visit compared with afternoon/evening meals provided [*F*(2,11) = 9.15–86.35, all *P* < 0.01, ƞ_p_^2^ = 0.45−0.89; Table 3]. Sedentary time was greater during the evening compared with morning/afternoon hours [*F*(2,11) = 60.75, *P* < 0.01, ƞ_p_^2^ = 0.85; Table 3]. Heart rate varied throughout the day, with higher values in the evening [*F*(2,11) = 9.94, *P* < 0.01, ƞ_p_^2^ = 0.47; Table 4]. End‐tidal carbon dioxide was greater during the morning compared with afternoon/evening hours [*F*(2,11) = 8.38, *P* < 0.01, ƞ_p_^2^ = 0.43; Table 4]. No differences in other resting haemodynamics were observed throughout the day (*F*‐value range, 0.27–2.31; *P* range, 0.12–0.76; ƞ_p_^2^ range, 0.02–0.17; Table 4). There was no effect of time of day on resting MCAv [*F*(2,11) = 0.69, *P* = 0.51, ƞ_p_^2^ = 0.06; Figure 1a or CvCi [*F*(2,11) = 0.96, *P* = 0.40, ƞ_p_^2^ = 0.08].

**TABLE 3:** Within‐day variability in lifestyle factors.

Factor	Morning (06.00–12.00 h)	Afternoon (12.00–18.00 h)	Evening (18.00–06.00 h)
Diet			
Total kilocalories	593 (560, 627)	535 (416, 653)	788 (690, 886)*, ^†^
Carbohydrates, %	61 (55, 68)	34 (30, 38)*	40 (37, 43)*
Fats, %	31 (26, 36)	42 (39, 45)*	36 (34, 39)
Proteins, %	9 (7, 11)	28 (26, 30)*	25 (24, 27)*
Physical activity, % daily activity			
Sedentary	53 (45, 60)	49 (43, 56)	80 (77, 84)*, ^†^
Low	27 (22, 33)	31 (26, 37)	11 (9, 13)*, ^†^
Low–moderate	11 (9, 14)	12 (10, 14)	6 (5, 7)*, ^†^
Moderate	7 (5, 8)	6 (5, 7)	3 (2, 4)*, ^†^
Moderate–vigorous	2 (1, 2)	1 (1, 2)	1 (0, 1)*, ^†^
Vigorous	0 (0, 0)	0 (0, 0)	0 (0, 0)

*Note*: Data are presented as the mean (95% confidence interval) from *n* = 12 participants. Data are averaged from two study visits (days 1 and 2) and were analysed using a one‐way repeated‐measures ANOVA or Friedman repeated‐measures ANOVA on ranks (% carbohydrates, % fats, vigorous).

* *P* < 0.05 versus morning.

^†^ *P* < 0.05 versus afternoon.

**TABLE 4:** Within‐day variability in resting haemodynamics.

Parameter	Morning (06.00–08.00 h)	Afternoon (12.00–14.00 h)	Evening (18.00–20.00 h)
Heart rate, beats/min	65 (61, 69)	65 (60, 69)	69 (65, 73)*, ^†^
Mean blood pressure, mmHg	83 (81, 85)	84 (82, 87)	86 (82, 89)
Respiratory rate, breaths/min	13 (11, 15)	13 (11, 15)	14 (12, 16)
Tidal volume, mL/breath	361 (290, 433)	345 (275, 416)	363 (315, 412)
Minute ventilation, L/min	4.5 (3.6, 5.4)	4.4 (3.6, 5.2)	4.8 (4.2, 5.5)
End‐tidal carbon dioxide, mmHg	44 (42, 46)	42 (41, 44)*	42 (40, 44)*
Middle cerebral artery velocity, cm/s	60 (53, 68)	58 (51, 65)	60 (53, 66)
Cerebrovascular conductance index, a.u.	73 (63, 83)	69 (60, 79)	71 (61, 80)

*Note*: Data are presented as the mean (95% confidence interval) from *n* = 12 participants unless otherwise noted. Data are averaged from two study visits (days 1 and 2) and were analysed using a one‐way repeated‐measures ANOVA.

* *P* < 0.05 versus morning.

^†^ *P* < 0.05 versus afternoon.

Heart rate [*F*(6,66) = 5.72, *P* < 0.01, ƞ_p_^2^ = 0.79], blood pressure [*F*(6,66) = 13.83, *P* < 0.01, ƞ_p_^2^ = 0.85], MCAv [*F*(6,66) = 52.33, *P* < 0.01, ƞ_p_^2^ = 0.96] and CvCi [*F*(6,66) = 30.15, *P* < 0.01, ƞ_p_^2^ = 0.94] increased during apnoea. The increase in blood pressure during apnoea was greater [*F*(2,22) = 9.27, *P* < 0.01, ƞ_p_^2^ = 0.74] and the increase in heart rate was lower [*F*(2,22) = 3.82, *P* = 0.04, ƞ_p_^2^ = 0.34] in the evening compared with morning/afternoon hours (Table 5). There was no effect of time of day on the MCAv [*F*(2,22) = 1.51, *P* = 0.24, ƞ_p_^2^ = 0.49] and/or CvCi response to apnoea [*F*(2,22) = 0.68, *P* = 0.52, ƞ_p_^2^ = 0.39; Table 5; see also Figure 1b,c].

**TABLE 5:** Within‐day variability in the haemodynamic response to 20 s end‐expiratory apnoea.

Parameter	Morning (06.00–08.00 h)	Afternoon (12.00–14.00 h)	Evening (18.00–20.00 h)
Heart rate, beats/min^a^, ^b^			
Baseline	66 (62, 70)	66 (62, 71)	69 (65, 74)
25%	76 (69, 82)	77 (71, 83)	79 (72, 85)
50%	73 (67, 79)	74 (68, 81)	76 (69, 82)
75%	71 (65, 78)	73 (66, 80)	74 (67, 81)
100%	70 (63, 77)	70 (63, 77)	72 (64, 79)
Recovery 50%	73 (68, 78)	72 (66, 78)	74 (68, 80)
Recovery 100%	67 (62, 73)	68 (62, 74)	71 (65, 77)
Mean blood pressure, mmHg			
Baseline	85 (83, 87)	85 (82, 87)	80 (76, 83)*, ^†^
25%	87 (85, 90)	85 (83, 88)	84 (80, 88)
50%	85 (81, 89)	84 (80, 88)	82 (77, 87)
75%	89 (84, 93)	88 (83, 93)	83 (78, 88)*, ^†^
100%	93 (88, 98)^‡^	92 (87, 98)^‡^	85 (79, 92)*, ^†^, ^‡^
Recovery 50%	97 (92, 102)^‡^	96 (91, 100)^‡^	88 (82, 94)*, ^†^, ^‡^
Recovery 100%	94 (89, 99)^‡^	92 (88, 95)^‡^	85 (80, 90)*, ^†^, ^‡^
Middle cerebral artery velocity, cm/s^a^			
Baseline	59 (52, 67)	57 (50, 64)	59 (52, 66)
25%	53 (46, 59)	51 (45, 56)	55 (49, 61)
50%	53 (46, 59)	49 (45, 54)	53 (48, 58)
75%	59 (52, 66)	57 (51, 62)	60 (54, 66)
100%	66 (58, 73)	63 (56, 69)	67 (60, 74)
Recovery 50%	71 (63, 78)	69 (62, 76)	71 (63, 79)
Recovery 100%	71 (64, 79)	69 (62, 76)	72 (64, 81)
Cerebrovascular conductance index, a.u.^a^			
Baseline	71 (61, 81)	68 (58, 77)	69 (60, 79)
25%	61 (53, 69)	60 (53, 67)	63 (55, 71)
50%	62 (54, 71)	60 (53, 66)	62 (54, 70)
75%	68 (58, 77)	66 (57, 74)	69 (59, 78)
100%	72 (61, 82)	69 (60, 78)	73 (63, 83)
Recovery 50%	75 (64, 85)	73 (63, 83)	76 (65, 87)
Recovery 100%	77 (67, 88)	76 (66, 86)	78 (67, 90)

*Note*: Data are reported as the mean (95% confidence interval) from *n* = 12 participants. Results are an average of four apnoea trials across two study visits (days 1 and 2) and were analysed using a two‐way repeated‐measures ANOVA.

^a^ Main effect of apnoea.

^b^ Main effect of time.

* *P* < 0.05 versus morning.

^†^ *P* < 0.05 versus afternoon.

^‡^ *P* < 0.05 versus baseline.

**FIGURE 2:**
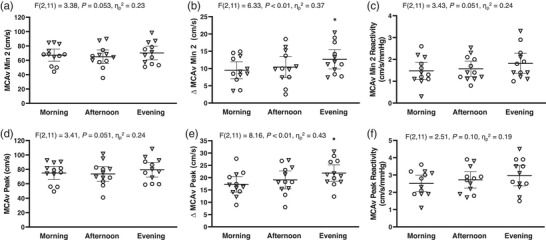
Diurnal variation in middle cerebral artery blood velocity (MCAv) during carbogen breathing. Data are presented as the mean and 95% confidence interval and as individual data points from *n* = 12 participants [six males (open circles) and six females (open inverted triangles)]. Statistical analysis was conducted using a one‐way repeated‐measures ANOVA. Diurnal variation in the MCAv response to carbogen exposure was observed when assessed as minute 2 average response (a–c) and peak response (d–f). ^*^ *P* < 0.05 versus morning.

Heart rate decreased [*F*(2,22) = 23.98, *P* < 0.01, ƞ_p_^2^ = 0.71] and blood pressure [*F*(2,22) = 20.76, *P* < 0.01, ƞ_p_^2^ = 0.68], end‐tidal carbon dioxide [*F*(2,22) = 184.28, *P* < 0.01, ƞ_p_^2^ = 0.95] and inspired oxygen [*F*(2,22) = 2024.82, *P* < 0.01, ƞ_p_^2^ = 0.99] increased during carbogen breathing (Table 6). The MCAv [*F*(2,22) = 62.47, *P* < 0.01, ƞ_p_^2^ = 0.96] and CvCi [*F*(2,22) = 56.72, *P* < 0.01, ƞ_p_^2^ = 0.95] increased during carbogen breathing (Table 6). Diurnal variation in the MCAv response to carbogen exposure was observed when assessed during minute 2 (Figure 2a–c) and as a peak response (Figure 2d–f).

**TABLE 6:** Within‐day variability in the haemodynamic response to carbogen breathing.

Parameter	Morning (06.00–08.00 h)	Afternoon (12.00–14.00 h)	Evening (18.00–20.00 h)
Heart rate, beats/min^a^, ^b^			
Baseline	66 (61, 70)	67 (63, 71)	70 (66, 75)
Min 1	65 (61, 69)	67 (62, 71)	69 (65, 73)
Min 2	63 (59, 67)	64 (60, 68)	67 (62, 71)
Mean blood pressure, mmHg^a^			
Baseline	86 (84, 89)	85 (83, 88)	86 (83, 89)
Min 1	86 (83, 89)	85 (82, 88)	87 (84, 91)
Min 2	88 (85, 91)	88 (85, 90)	89 (86, 92)
End‐tidal carbon dioxide, mmHg^a^			
Baseline	42 (40, 44)	42 (40, 43)	42 (40, 43)
Min 1	46 (45, 48)^‡^	46 (45, 47)^‡^	47 (45, 48)^‡^
Min 2	49 (48, 50)^‡^, ^§^	48 (47, 49)^‡^, ^§^	49 (48, 50)^‡^, ^§^
Inspired oxygen, mmHg			
Baseline	149 (147, 152)	149 (146, 152)	149 (146, 152)
Min 1	563 (523, 602)^‡^	586 (563, 609)^‡^	608 (592, 624)* ^,^ ^‡^
Min 2	585 (559, 612)^‡^	600 (584, 615)^‡^	617 (606, 629)* ^,^ ^‡^
Middle cerebral artery velocity, cm/s			
Baseline	58 (50, 65)	55 (47, 62)	58 (50, 65)
Min 1	61 (54, 69)^‡^	58 (51, 66)^‡^	63 (55, 70)^‡^
Min 2	67 (60, 75)^‡^, ^§^	65 (56, 73)^‡^, ^§^	70 (62, 79)^†^, ^‡^, ^§^
Cerebrovascular conductance index, a.u.^a^			
Baseline	67 (58, 76)	64 (55, 73)	67 (57, 77)
Min 1	72 (61, 82)	69 (60, 78)	72 (62, 82)
Min 2	77 (67, 87)	75 (65, 84)	80 (69, 91)

*Note*: Data are reported as the mean (95% confidence interval) from *n* = 12 participants. Data are averaged from two study visits (days 1 and 2) and were analysed using a two‐way repeated‐measures ANOVA.

^a^ Main effect of carbogen.

^b^ Main effect of time.

* *P* < 0.05 versus morning.

^†^ *P* < 0.05 versus afternoon.

^‡^ *P* < 0.05 versus baseline.

^§^ *P* < 0.05 versus minute 1.

**FIGURE 3:**
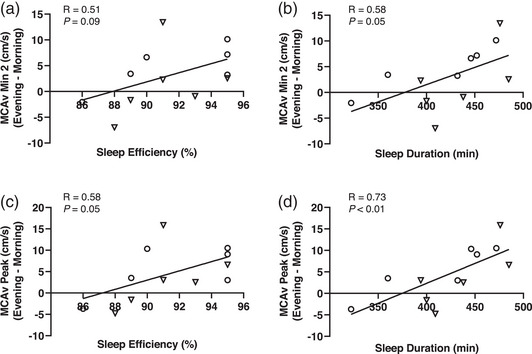
Diurnal variation in middle cerebral artery blood velocity (MCAv) and sleep. Data are presented as individual data points from *n* = 12 participants [six males (open circles) and six females (open inverted triangles)]. Statistical analysis was conducted using linear regression analysis and Pearson product–moment correlation. Diurnal variation (evening minus morning) in minute 2 (a,b) and peak (c,d) MCAv response to carbogen are correlated with sleep efficiency and duration.

**FIGURE 4:**
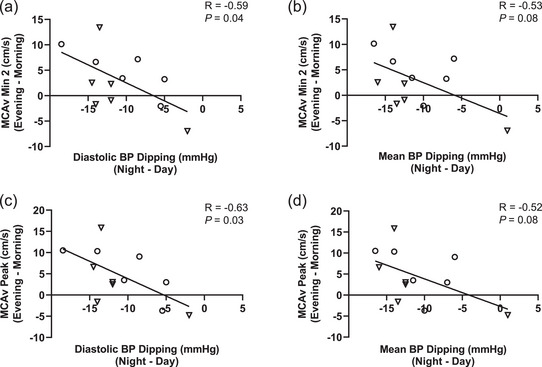
Diurnal variation in middle cerebral artery blood velocity (MCAv) and nocturnal blood pressure dipping. Data are presented as individual data points from *n* = 12 participants [six males (open circles) and six females (open inverted triangles)]. Statistical analysis was conducted using linear regression analysis and Pearson product–moment correlation. Diurnal variation (evening minus morning) in minute 2 (a,b) and peak (c,d) MCAv response to carbogen are correlated with diastolic, and mean nocturnal blood pressure dipping.

In a post hoc analysis, the response relationship measure of diurnal variation of MCAv during carbogen breathing and participant characteristics (e.g., age, body mass index and chronotype) were explored using linear regression analysis and Pearson product–moment correlation. Diurnal variation in both minute 2 and peak MCAv during carbogen breathing (evening minus morning) were found to be correlated with sleep efficiency (minute 2, *r* = 0.51, *P* = 0.09; peak, *r* = 0.58, *P* = 0.05; Figure 3a,c), sleep duration (minute 2, *r* = 0.58, *P* = 0.05; peak, *r* = 0.73, *P* < 0.01; Figure 3b,d), diastolic (minute 2, *r* = −0.59, *P* = 0.04; peak, *r* = −0.63, *P* = 0.03; Figure 4a,c) and mean (minute 2, *r* = −0.53, *P* = 0.08; peak, *r* = −0.52, *P* = 0.08; Figure 4b,d) blood pressure dipping. No other significant correlations were observed.

## 4 DISCUSSION

The main findings from the present investigation are threefold. First, we show that measures of MCAv assessed during morning, afternoon and evening hours are reproducible between days in a mixed cohort of male and female participants. Second, we observed diurnal variation in the MCAv response to carbogen exposure, with values highest during evening compared with morning hours. Third, we found that longer, more efficient sleep and greater nocturnal blood pressure dipping are associated with greater diurnal variation in the MCAv response to carbogen in young, healthy adults. These data enhance our understanding of potential modulators of MCAv and CVR and have important implications for translation to clinical practice.

Diurnality of cerebrovascular haemodynamics has been hypothesized to contribute to cerebral ischaemia and stroke, which occur predominantly in the morning hours (Elliott, 1998). Herein, we show that there is no diurnal variation in resting MCAv or CvCi (Figure 1; Table 4) in healthy young male and female adults when potential confounding factors (e.g., physical activity, meals) are tightly controlled. These data agree with prior work (Cummings et al., 2007; Strohm et al., 2014) showing that resting MCAv is relatively stable throughout the day. However, these results are in contrast to data from Demolis et al. (1993), in addition to others (Conroy et al., 2005; Diamant et al., 2002; Lewis et al., 2011), who identified diurnal variation in resting MCAv. Discrepancies between findings might be attributable to study conditions, such as completing measurements in the fed versus fasted state (Conroy et al., 2005; Demolis et al., 1993), and to differences in the age (Diamant et al., 2002) and/or sex [majority male; (Conroy et al., 2005; Demolis et al., 1993; Lewis et al., 2011)] of participants in prior studies. Upon closer examination of the present data, we found that resting MCAv was consistently higher in young, healthy premenopausal female participants when compared with male adults, with no sex‐related differences in diurnal variation. Although few have examined diurnal variation in MCAv, many groups have also shown sex‐related differences in cerebrovascular regulation (Aanerud et al., 2017; Alwatban et al., 2021; Matteis et al., 1998; Tegeler et al., 2013). All female participants in the present investigation had natural menstrual cycles (i.e., were not prescribed oral hormonal contraceptive pills) and were studied during the early follicular (low‐hormone) phase of the menstrual cycle owing to known modulatory effects of oestrogen and progesterone on cerebral blood flow (Cote et al., 2021; Nevo et al., 2007). With this, the present investigation strengthens prior results and ultimately supports that diurnal variation in resting MCAv is unlikely to be observed in healthy young male or female adults when experimental controls are present.

Importantly, we show that a lack of diurnal variation in resting MCAv from the combined cohort of males and females is unlikely to be attributable to a lack of reproducibility in the measure. Using a variety of statistical and analytical approaches, main findings show moderate to good reproducibility and low between‐day differences in MCAv measured in the resting state (Table 2). This confirms previous results showing good to excellent reproducibility of MCAv measurements collected during morning hours (06.00–10.00 h). Unfortunately, the majority of prior data assessing the reproducibility of MCAv measures using TCD, both at rest (Baumgartner et al., 1994; Demolis et al., 1993; Maeda et al., 1990; Totaro et al., 1992) and in response to a physiological challenge (e.g., apnoea, carbogen) (Koep et al., 2022; Mayberg et al., 1996; McDonnell et al., 2013; Totaro et al., 1999), have been conducted in the morning hours only. From those studies that have assessed reproducibility of TCD data in the evening, moderate to good reproducibility in the MCAv response to carbon dioxide has been reported (Cummings et al., 2007; Strohm et al., 2014). We expand these data and show moderate to good reproducibility in measures of MCAv both at rest or in response to apnoea and/or carbogen breathing throughout the day. With this, it is important to acknowledge that the reproducibility of the cerebral haemodynamic response to carbogen is preferrable to that observed in response to apnoea, especially during the afternoon hours, and reporting of absolute MCAv (in centimetres per second) rather than CVR (in centimetres per second per millimetre of mercury) is recommended owing to poor reproducibility of CVR in the afternoon/evening observed herein (Table 2). Poor reproducibility during apnoea might be attributable, in part, to varied carbon dioxide levels (Fierstra et al., 2013; Hoiland et al., 2019; Tancredi & Hoge, 2013; Totaro et al., 1999), large changes in blood pressure caused by volitional stress (Regan et al., 2014) and incorrect performances of a breath hold, subsequently leading to underestimation of CVR (van Beek et al., 2011). Furthermore, the present study was conducted in free‐living conditions to enhance translation. Although speculative, differences in daily activities (e.g., classwork and examinations) intrinsic to the free‐living conditions, particularly in college‐aged adults, might have contributed to an increase in the variability of MCAv measurements in the afternoon, when such activities might be more likely to occur.

Despite a lack of diurnal variation in the response to apnoea, significant differences in the MCAv response to carbogen breathing throughout the day were noted; specifically, MCAv during carbogen breathing was highest in the evening compared with morning (Figure 2) and afternoon (Table 6) hours. Diurnal variation in resting MCAv has been proposed primarily to be nitric oxide (NO) mediated, such that inhibition of NO synthase with N(omega)‐nitro‐L‐arginine (L‐NNA) in lambs prevents sleep–wake differences (Zoccoli et al., 2001). However, the cerebrovascular response to carbon dioxide in humans is relatively unaffected by inhibition of NO synthase (Hoiland et al., 2022; White et al., 1998). With this information in mind, mechanisms contributing to diurnal variation in CVR in humans are likely to be varied. For example, fragmented sleep (Qureshi et al., 1999) and overnight reductions in flow‐mediated vasodilatation (Ainslie et al., 2007) have been associated with a diminished hypercapnic vasomotor response in the morning. Others have shown MCAv to exhibit 24 h rhythmicity independent of external stimuli (including sleep), supporting an independent effect of circadian influences (Conroy et al., 2005). To gain a better understanding of the factors that might contribute to diurnal variation in CVR, we explored correlations between measures of diurnal variation in MCAv and indices of sleep health. Notably, although participants were instructed to sleep for 7–9 h per night, objective monitoring indicated non‐adherence [5 of 12 (42%)] to sleep guidelines. With these data, we found that those individuals with greater sleep efficiency, sleep duration and nocturnal ‘dips’ in blood pressure exhibited greater diurnal variation in the MCAv response to carbogen exposure (Figures 3 and 4). These results are in contrast to prior work in middle‐aged adults, where greater blood pressure dipping was associated with higher resting MCAv and greater reactivity to carbon dioxide in the morning (09.00 h) (Hajjar et al., 2007). Differences between study cohorts [participants were older (average 54 years), 45% had clinical hypertension, and 66% were considered ‘non‐dippers’ (nighttime blood pressure decline by <10%)] probably contributed to the discrepancies between results.

### 4.1 Experimental considerations

Strengths of the present investigation include multiple within‐day experimental visits, in addition to comparisons between days to assess both diurnal variation and reproducibility of haemodynamic measurements within the same mixed‐sex cohort. Additionally, the controlled nature of the study (i.e., sleep, physical activity and meals), which includes studying female participants with natural menstrual cycles during the early follicular phase, limit the influence of cofounding factors on MCAv and CVR outcomes. Although the present study provides important considerations and expands on previous work assessing MCAv and CVR, there are a few limitations to acknowledge. First, participants were exposed to 2 min of carbogen breathing. Recent data suggest that the peak MCAv response to carbon dioxide might not be achieved in all individuals within 2 min (i.e., ‘slow responders’) (Burley et al., 2020). However, a strength of the shorter protocol duration includes limiting the potential effect of prolonged carbon dioxide exposure on vessel diameter (Al‐Khazraji et al., 2019) and minute ventilation (Carr et al., 2021). With this, future studies combining intracranial and extracranial measures and protocols extended to 3 min (Burley et al., 2020) might provide greater insight. Future studies should also consider the influence of an overnight fast and hunger levels on morning measurements, because hunger might affect cerebral haemodynamics (Wierenga et al., 2017). Additionally, measurements were made in healthy, young and mostly Caucasian adults. It is interesting to speculate whether differences would be observed in other populations. This is especially true considering recent data supporting sex and racial differences in the effect of varying sleep quality (Johnson et al., 2019) and blood pressure dipping (Booth et al., 2019) on peripheral vascular health.

## 5 CONCLUSION

The present study fills a key gap in knowledge and indicates that MCAv measurements taken in the morning, afternoon and evening hours exhibit diurnal variation and are reproducible between days in healthy young male and female participants. Additionally, greater sleep efficiency and nocturnal blood pressure dipping are correlated with greater diurnal variation in the MCAv response to carbogen. These findings advance our understanding of potential modulators of MCAv and CVR and improve translation to real‐life application.

## AUTHOR CONTRIBUTIONS

Experiments were conducted in the laboratory of Jacqueline Limberg at the University of Missouri. Brian Shariffi and Jacqueline Limberg conceived and designed the work. Brian Shariffi, Iman Lloyd, Mikala Cessac, Jennifer Harper and Jacqueline Limberg were involved in the acquisition, analysis and interpretation of the data. Brian Shariffi and Jacqueline Limberg drafted the manuscript. Brian Shariffi, Iman Lloyd, Mikala Cessac, Jennifer Harper and Jacqueline Limberg revised the manuscript critically for important intellectual content. All authors approved the final version of the manuscript and agree to be accountable for all aspects of the work in ensuring that questions related to the accuracy or integrity of any part of the work are appropriately investigated and resolved. All persons designated as authors qualify for authorship, and all those who qualify for authorship are listed.

## CONFLICT OF INTEREST

None declared.

## Supporting information


Statistical Summary Document

## Data Availability

The data that support the findings from the present study are available from the corresponding author upon reasonable request.
